# Gaze Position Reveals Impaired Attentional Shift during Visual Word Recognition in Dysfluent Readers

**DOI:** 10.1371/journal.pone.0108937

**Published:** 2014-09-30

**Authors:** Jarkko Hautala, Tiina Parviainen

**Affiliations:** 1 Agora Center, University of Jyväskylä, Jyväskylä, Finland; 2 Centre for Interdisciplinary Brain Research, Department of Psychology, University of Jyväskylä, Jyväskylä, Finland; University of Leicester, United Kingdom

## Abstract

Effects reflecting serial within-word processing are frequently found in pseudo- and non-word recognition tasks not only among fluent, but especially among dyslexic readers. However, the time course and locus of these serial within-word processing effects in the cognitive hierarchy (i.e., orthographic, phonological, lexical) have remained elusive. We studied whether a subject's eye movements during a lexical decision task would provide information about the temporal dynamics of serial within-word processing. We assumed that if there is serial within-word processing proceeding from left to right, items with informative beginnings would attract the gaze position and (micro-)saccadic eye movements earlier in time relative to those with informative endings. In addition, we compared responses to word, non-word, and pseudo-word items to study whether serial within-word processing stems mainly from a lexical, orthographic, or phonological processing level, respectively. Gaze positions showed earlier responses to anomalies located at pseudo- and non-word beginnings rather than endings, whereas informative word beginnings or endings did not affect gaze positions. The overall pattern of results suggests parallel letter processing of real words and rapid serial within-word processing when reading novel words. Dysfluent readers' gaze position responses toward anomalies located at pseudo- and non-word endings were delayed substantially, suggesting impairment in serial processing at an orthographic processing level.

## Introduction

Previous research of visual word recognition has relied mostly on response times. As end-point measures, response times cannot inform us about the time course of cognitive processing. In this study, we explore whether miniature eye movements [1], [2], [3] reflect word recognition processes as they unfold in time in fluent and dysfluent readers. This working hypothesis is based on evidence that the spatial distribution of lexical information within a word affects the landing position of an initial saccade made during continuous reading; studies by Hyönä and colleagues [4]–[5] provided direct evidence that the initial fixation position was pulled toward the start of beginning-informative words. Furthermore, word refixations are generally progressive (i.e., oriented toward reading direction), suggesting the presence of serial shifts of attention during the reading of a word. We hypothesized that even the smallest eye movements may be affected by this serial within-word processing, at least when reading pseudo- and non-words.

Indeed, some of the miniature eye movements during fixation are found to reflect attentional processes. During fixations, the eyes perform micromovements such as microsaccades, tremors, and drifts [6], which serve vision by continuously stimulating the retinal cells [7], [8]. Research conducted about attentional cueing paradigms (a subject that focuses on a fixation cross and reports in which direction a spatial cue was presented) has shown that involuntary and ballistic microsaccades are an index of covert visual attention [1], [9]. As reviewed by Martinez-Conde [10], microsaccades and saccades seem to be physiologically indistinguishable: they can both be involuntary and voluntary [11], and when microsaccades occur during reading, they seem to be refixations overlapping due to their amplitude with microsaccades and triggered in conjunction with the saccadic rate, that is, not affecting the overall rate of eye movements [12]. However, during reading, the saccadic rate (including refixations) is much higher, around 4 hz, relative to the microsaccade rate in attentional cueing paradigms (0.2–2 hz). In addition to microsaccades, drifting may be partially governed by cognitive processing [2], so we will also analyze the raw gaze position signal.

So far, only one study has explored the orthographic influences on micromovements during reading [3]. The data revealed an underlying tendency to hold the eyes still during natural reading fixations, except when reading text with alternating case, for which micromovements were oriented more frequently to the right than the left. This result is in line with the view that serial within-word processing occurs when reading novel words. Very few studies have explored saccadic eye movements during visual word recognition. Kuperman and Bertram [13] studied eye movements during complex compound word-processing in a lexical decision task. Along with more fine-grained analyses of compound word-processing, they also reported a right-ward progression of saccades as typically seen in eye movements during text reading.

Generally speaking, visual word recognition involves a sequence of visual, orthographic, phonological, and semantic processes [14]–[17]. The location and timing of the pathway in the brain underlying these processes have been indicated by functional neuroimaging studies to proceed from occipital sensory areas to the language-related cortex in the left (and right) temporal lobes. Visual features of words are processed from ∼100 ms after word onset, followed by the processing of orthographic features from 150 ms onwards [18]–[22], sublexical orthography-to-phonology mapping from 200 ms onwards [23], and, finally, lexico-semantic processing peaking at 400 ms after stimulus onset [24]–[29]. Within this sequence, both serial decoding and holistic word recognition seem to occur [30].

Serial decoding of letters is crucial for early reading development and for reading novel words; repeated exposure to the same words is assumed to strengthen orthographic word representations, thus providing a basis for whole-word recognition [31]–[35]. Readers with developmental dyslexia show prolonged reliance on serial decoding even in real-word reading [36]–[39] and seem to attain whole-word recognition skills only in adolescence [40], [41]. Moreover, adult readers with dyslexia seem to be substantially slow in reading long pseudo-words [40], which suggest a permanent inefficiency in the phonological, serial reading procedure. Brain imaging studies of visual word recognition in readers with dyslexia show diminished activation for letter strings at around 150 ms in the occipitotemporal cortex [42], [43], [19], suggesting deficient orthographic processing and increased activation in the frontal areas [43], presumably reflecting increased reliance on articulatory decoding [43]. Taken together, slow and effortful reading by dyslexic people seems to reflect non-optimal applications of serial, grapheme-to-phoneme decoding.

For studying spatially selective eye-movement responses during word decoding, one needs to manipulate some property of the sublexical units of words in a spatially selective manner, such as varying the frequency of sublexical units located at word beginnings or endings. We manipulated the *uniqueness point (UP)* of words and *deviation point (DP)* of the non-word and pseudo-word analogues. UP refers to the letter position at which a word differentiates itself from all other words in a language, and non-word DP refers to the letter position at which the item no longer matches any real word; that is, no other word (or no word, in the case of pseudo- and non-words) in the given language has that same beginning [44], [45]. From the serial decoding point of view, a unique word beginning could be used as a cue for identifying words, thereby facilitating the lexical search. In contrast, if all letters are processed in parallel, the UP/DP should have no effect on word recognition. Empirically, response times for early UP words are generally faster than those for late UP words in naming [44], [45] and lexical decision tasks [46], whereas this effect was not found in readers' eye movements during natural reading [47]. In one study, late DPs were found to trigger a faster response than early DPs [45]. All of these previous studies were conducted in the opaque English orthography, for which mapping between graphemes and phonemes is not straightforward. In such orthography, letters appearing later in a word affect the pronunciation of earlier letters. This reduces the feasibility of a serial decoding strategy. Instead, the present study is conducted in the fully transparent Finnish orthography, with one-to-one correspondence between letters and phonemes. In such transparent orthography, serial sublexical decoding (either in letters, bigrams, trigrams, syllables or morphemes) is a potent reading strategy, and serial effects during reading are frequently observed [36]–[42].

For detecting the time course of serial within-word processing, we will study the point in time at which early and late DP starts to pull the reader's gaze toward the deviation. From the serial within-word processing perspective, we expect an early UP/DP to attract the gaze earlier in time than a late UP/DP. The temporal gap between the effect of early and late UP/DP would provide a rough estimation of the speed of the serial process. Dysfluent readers are expected to show a pattern of results compatible with slower serial within-word processing in pseudo- and non-word reading relative to fluent readers. In eye movements, this would be reflected by an increased temporal gap between the effects of early and late DPs.

For exploring the processing levels in the cognitive hierarchy at which the serial processing mainly occurs, we compare eye-movement responses to UP/DP manipulation in different types of items—in real words, pseudo-words, and non-words. It is assumed that real words can be rapidly recognized based on orthographic memory representations, and non-words can be rapidly detected due to infrequent initial or final bigrams. However, word-like pseudo-words are expected to be the subject of prolonged decoding or lexical search processes. Based on a dual-route view of reading [14], [16], [17], we expect that fixational eye-movement responses reflecting serial within-word processing are more likely to be found with pseudo- and non-word stimuli than with real-word stimuli.

## Methods

### Ethics statement

The research was conducted according to the principles expressed in the Declaration of Helsinki. Prior to the research, ethical approval was obtained from the ethical committee of the University of Jyväskylä. Informed written consent from the subjects and the caregivers of underage subjects was obtained prior to the research; all the data were analyzed anonymously, and personal information was handled confidentially.

### Subjects

The subjects included 35 native Finnish-speaking persons aged 16 to 36 years. They were recruited from upper secondary and higher education institutions in the Jyväskylä region using the following screening procedure: An email message was distributed via school administration to all students. Students who were interested completed a web-delivered contact information query and a short screening task (LUKSU) [48] for reading fluency, adopted from reading fluency subtasks in Woodcock-Johnson III Tests of Achievement [49]. In the self-paced LUKSU task, subjects read a sentence and decided if it was sensible or not by clicking a mouse button. The subject's score was determined by the number of correct answers given within the one-minute time limit. Participants were divided into two groups based on their scores. Those who scored in the lowest quartile were invited to participate in the study for potential dysfluent readers (without it being identified as such). Subjects who performed above the lowest quartile were invited to participate in the study for potential control subjects (also not identified as such).

During the research visit, the subjects' reading skills were further assessed using the Assessment Battery for Reading Disabilities in Young and Adults [50], from which they completed subtasks on standardized word reading, pseudo-word reading, and text reading. The IQs of the participants were assessed via the Raven's Standard Progressive Matrices [51], administered without a time limit. In addition, participants completed tests involving rapid automatized naming (RAN), rapid alternating series (RAS), and a Finnish version of the phonological spoonerism task. Table 1 features a summary of the participants' performances in these tasks along with the results of independent sample *t* tests between the groups.

**Table 1:** Demographic data of Age, Reading and Related Skills, and Cognitive Ability for Fluent and Dysfluent Reader Groups.

	Fluent (N = 20)		Dysfluent (N = 12)		t-test	
	Mean	SD	Mean	SD	t(31)	p
Age in years	19.35	3.99	24.41	5.99	−2.87	.007
Raven SPM, score max 60	54.10	3.67	52.83	4.45	.873	.389
Word list, time in sec	22.66	5.17	36.04	6.03	−6.66	.000
Pseudo-word list, time in sec	42.96	8.93	69.22	13.99	−5.83	.000
Text reading, words in 3 min	362.3	36.07	296.0	22.44	5.71	.000
Word list accuracy	99.3%	.01%	97.7%	.03%	1.72	.109
Pseudo-word accuracy	88.7%	.08%	81.1%	.12%	1.62	.122
Text reading accuracy	99.3%	.74%	98.9%	.58%	1.42	.166
Spoonerism, max 15	11.75	3.53	8.83	5.30	1.69	.109
RAS, time in sec	28.65	4.19	39.36	8.99	−3.88	.000
RAN, time in sec	36.05	6.44	40.68	8.37	−1.72	.096

Participants were considered to be dysfluent readers (DYS) if, despite average IQ scores, they belonged to the lowest 11 percentage points, according to standardized test scores, either in the text or word-reading tasks. To be considered a fluent reader (FLUENT), participants had to perform 11 percentage points above the population standards in all of the reading tasks. These criteria led to the exclusion of two subjects. In addition, we excluded data from three subjects, one due to technical problems and two due to the subjects' strong corrective lenses having prevented the system from registering eye movements. Two subjects showed an abnormal distribution of (micro-)saccades, and their results were excluded from the analysis. In total, there were 32 response time data sets, 30 gaze position data sets, and 28 (micro-)saccade data sets.

### Equipment

The lexical decision task was administered by the E-Prime 1.0 program running on a standard desktop computer. Right-eye movements were registered by the SMI HiSpeed iViewX eye tracker at a sampling rate of 500 Hz. The subjects leaned on the forehead and chin rests of an eye-tracking column at a viewing distance of 67 cm.

### Materials

The stimuli consisted of 240 pseudo/non-words and 240 words (nouns). Both sets included 80 five-letter items, 80 six-letter items, and 80 seven-letter items. Each four pseudo- and non-word category (early- and late-deviating pseudo and non-words) contained 48 items (16 of each item length). In addition, there were 48 consonant strings excluded from the analyses. In addition, some of the pseudo-words were dropped post-hoc from the analyses for controlling initial bigram frequencies (See Footnote 2 of Table 2). Among the five- and six-letter words, there were 40 items with an early UP and 40 items with a late UP. UP manipulation in seven-letter words was omitted due to the redundant endings of longer words in Finnish (e.g., *pal* ***ikka*** (block) and *pen* ***ikka*** (pup)). See Table 2 for descriptive statistics of the stimuli included in the analyses.

**Table 2:** Description of subset of experimental stimuli selected for the analysis.

Item type		Words^1^			Pseudo-words^2^			Non-words		
Uniqueness/Deviation point class		Early	Late	*p*	Early	Late	*p*	Early	Late	*p*
Number of Items		40	40		39	39		48	48	
Average DP/UP (in letters)		3.60	5.48	**	3.72	6.00	**	2.55	5.94	**
Bigrams (in thousand words)	Init.	8.48	14.30	**	11.2	13.3	ns.	.20	10.20	**
	Mid.	8.64	11.20	*	9.95	9.61	ns.	9.42	9.34	ns.
	Final	9.92	8.96	ns.	8.72	6.99	ns.	9.19	.07	**
Word or base-word frequency (in million words)		11.0	8.5	ns.	20.0	14.2	ns.	25.4	14.8	ns.

ns.>.05,

* *p*<.05,

** *p*<.001.

1 Due to strong manipulation of word uniqueness points, words with unique beginnings also had somewhat lower initial bigram frequencies.

Despite these two potential gaze-attracting factors, no word uniqueness point was observed in eye movement measures.

2 For ensuring that a pseudo-word deviation point effect does not partly result from orthographic processing, initial and final bigram frequencies between early and late deviating pseudo-words were controlled with a post-hoc procedure, in which 18 pseudo-words having the lowest initial or final bigram frequency were excluded from the analyses.

Bigram frequencies are expressed as occurrences per 1000 words.

Based on a frequency corpus of modern written Finnish words [52], UPs and DPs were determined manually, and sublexical frequencies were calculated. We excluded corpus items with less than five occurrences and surnames without semantic meanings.

The word items were then used to generate the pseudo- and non-word items. The pseudo-words with early and late DPs were constructed by replacing either the first or last letter in a word with another Finnish letter in such a way that a phonotactically valid Finnish pseudo-word was formed. For example, for the base word *kirkko* (church), early and late DP pseudo-words could be *rirkko and kirkki*, respectively. The non-words were constructed in a similar manner, but the replacement-letter was chosen among foreign-origin letters (*q*, *w*, *d*, *f*, *g*, *z*, *x*, *c*, *b*) to form non-words with highly infrequent, nonexistent or ill-formed word beginnings and endings (e.g., *qirkko* and *kirkkq*).

### Procedure

At the start of each trial a black cross on a white screen was displayed horizontally in the middle of the screen and vertically at 25% from the upper frame of the screen. After 1000 ms the experimental item was presented in the same location. The horizontal midpoint of each word was located at the center of the screen's *x* axis. The participants' task was to decide whether the item was an existing Finnish word or not. They registered their decision by clicking the left or the right mouse button, with the assignment of correct and incorrect response buttons counterbalanced among subjects. Items were presented in monospaced 24-point Courier New font with each letter corresponding to 0.57 visual degrees. The item remained on the screen until a response was registered. A blank screen appeared for 1000 ms between the trials. After reading written instructions, the subject completed a practice run of eight trials followed by an eye-tracker calibration (13-point calibration with strong check level). They viewed items of different numbers of letters in separate blocks (160 items), counterbalanced across participants with the presentation order of the items within each block randomized for each subject. The blocks were interrupted by short pauses during which the eye tracker was recalibrated.

### Data processing

#### Response time data

Out of 16,320 trials, 547 (3.3%) were responded to incorrectly, with DYS readers performing at 95.2% and FLUENT readers at 97.4% accuracy rates. In addition, 413 trials (2.9%) were responded to with a response time that was slower or faster than 2.5 standard deviations from the subject mean; these were excluded from the analyses.

#### Gaze position data

As the gaze remained still for the first 150 ms after stimulus presentation, the first 0–100 ms time window was the baseline to correct for variations in the calibration accuracy of gaze position data. For each trial, the raw *x* coordinate gaze position data were first aggregated to 10 ms time bins. Then, data points deviating more than 2.5 visual degrees (80 pixels, 4.5 letter spaces, 0.2% of all cases) from the baseline were excluded and the data was further averaged to 40 ms time bins, resulting in 12 time-windows.

#### Saccade and microsaccade detection

**Figure 1:**
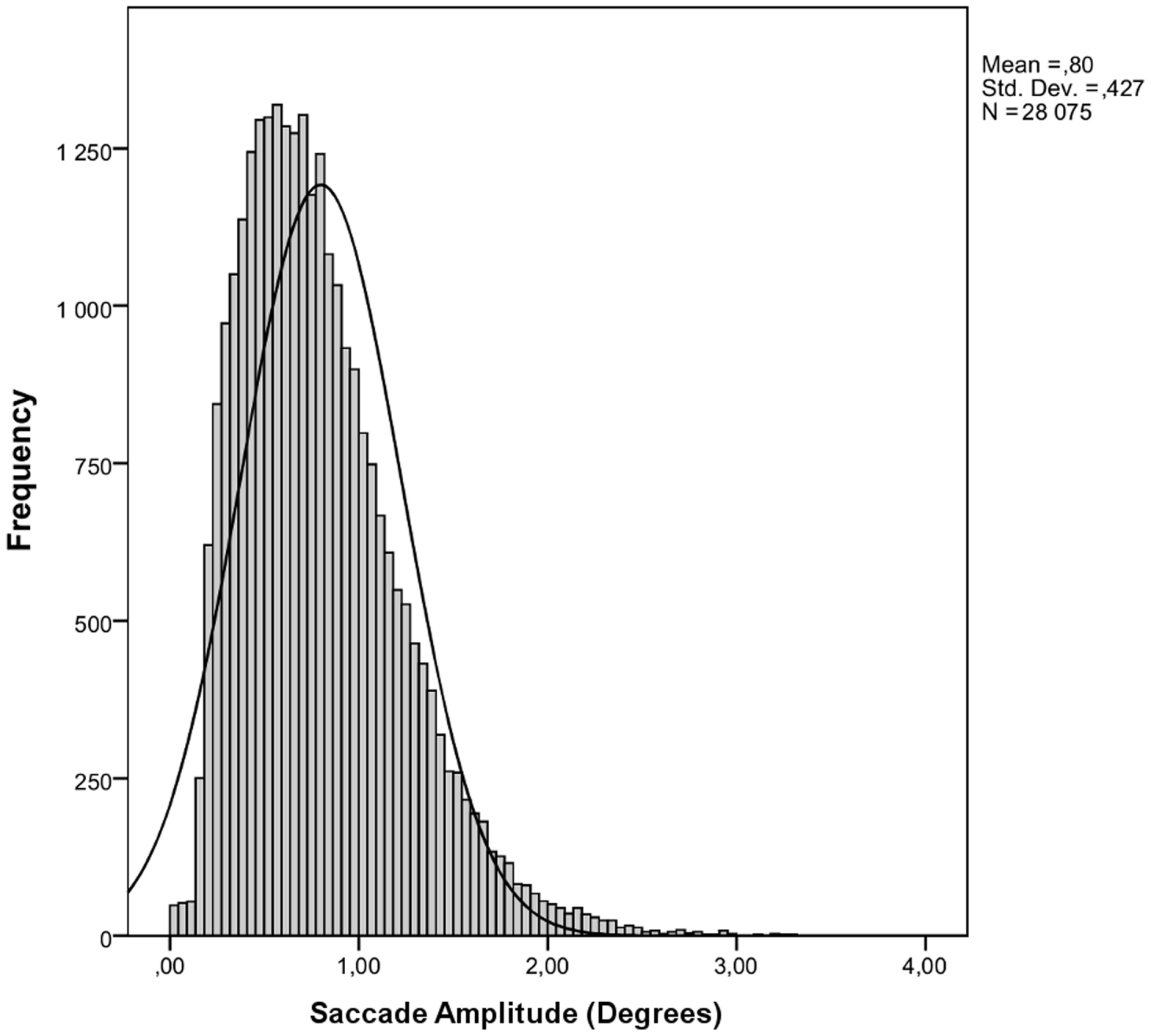
Distribution of saccade amplitudes.

**Figure 2:**
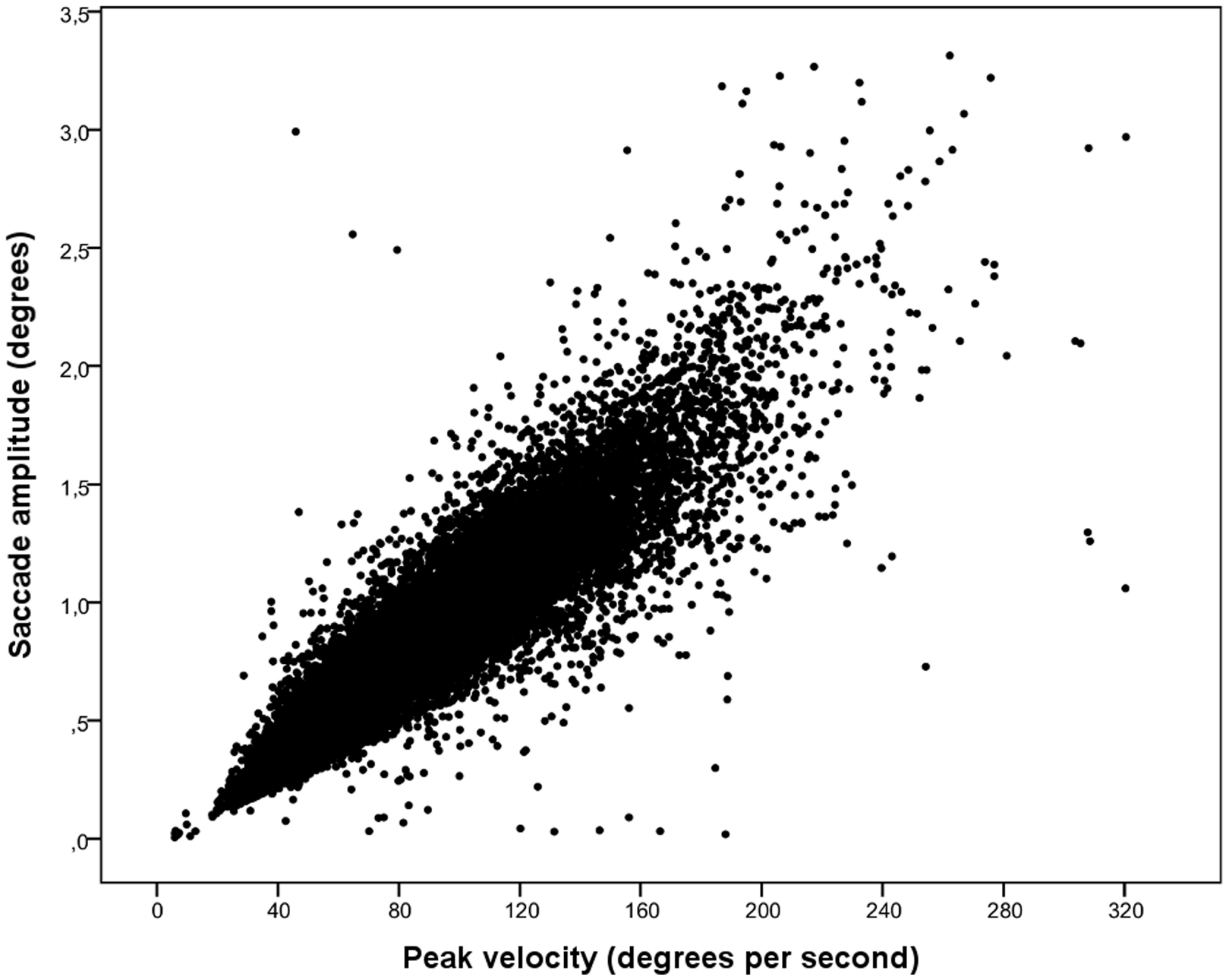
Saccade amplitudes as a function of peak velocities.

**Figure 3:**
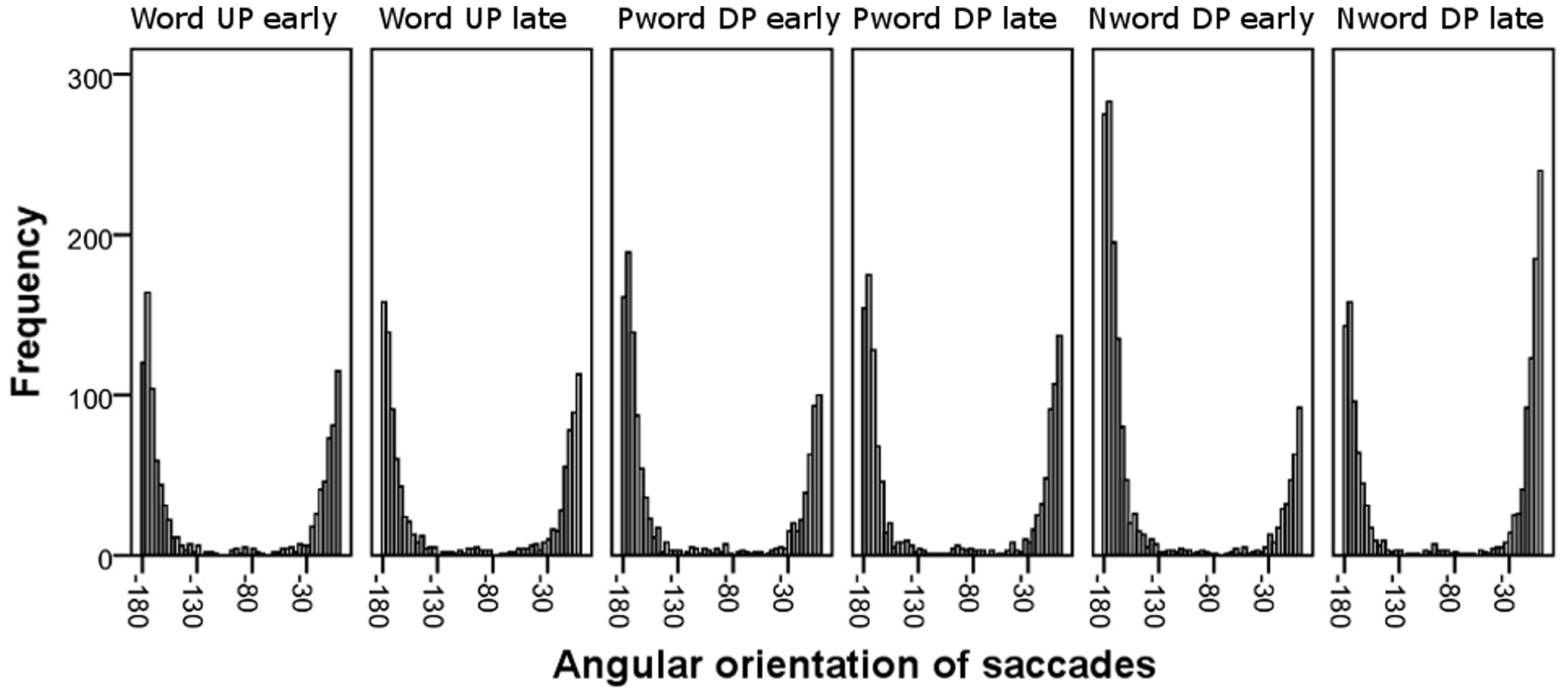
The spatial distribution of saccade orientation on a horizontal axis. The values range from 0 (right) to -180 (left) directions.

**Figure 4:**
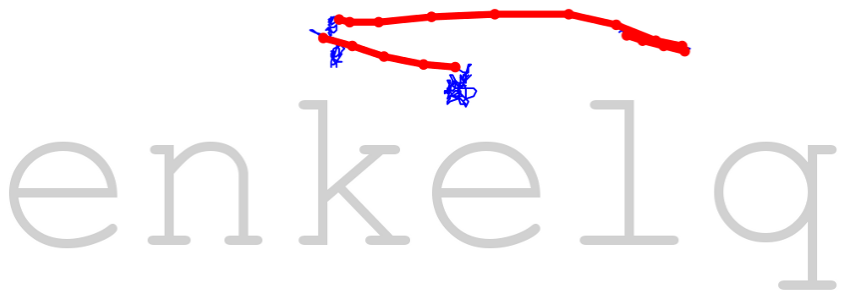
Plotted data from a single trial horizontally aligned with stimuli presented. Non-saccadic data points shown in blue, saccadic data points shown in red.

For microsaccade and saccade detection, we applied an algorithm developed by Engbert and Kliegl [1] with the following parameters: a minimum duration of four samples (8 ms) and a velocity threshold of eight standard deviations counted from a moving average of six successive samples. Due to monocular recording, it was not possible to apply the binocular criteria of microsaccades. Compensatively, and to avoid noises that might be detected as saccades, we used a somewhat higher saccade velocity threshold than typically used in microsaccade studies. We excluded the data of two subjects from the analyses due to the aberrant distribution of saccade peak velocity and amplitude. To exclude corrective saccades typically occurring instantly after a saccade, that is, glissades, we applied an intersaccade interval criterion of longer than 50 ms, leading to the exclusion of 16.5% of all cases. Extreme saccades with a peak velocity higher than 333°/s (7.2% of all cases) and an amplitude higher than 3.3 degrees (6.7% of all cases) were discarded. The saccade amplitude was calculated as the difference between the starting and ending location of a saccade. See Figures 1, 2, 3, 4 for descriptive information about the saccadic behavior during our task, and the Methodological considerations section at the end of the Discussion for issues of microsaccade definitions.

### Statistical analyses

We analyzed the response time, gaze position, and microsaccade data with repeated measures of ANOVAs for between-subject (*F1*) and between-item data (*F2*). Because we did not include UP manipulation in seven-letter words, the effect of UP could not be compared with DP effects. Therefore, we ran separate ANOVAs for real-word data and for pseudo- and non-word data. This means that, depending on the analysis, there was a two-level factor of item types (pseudo-words, non-words), a two-level factor of DP/UP (early, late), and a between-subject factor of reading fluency (fluent, dysfluent). In the gaze position analysis, we ran separate ANOVAs for each of the 12 time bins. In the between-subject analysis, the item type and DP/UP were within-subject factors. In the between-item analysis, the group was within-subject factors and the item type and DP/UP factors were between-subject factors. Dependent variables included response times in milliseconds, gaze position (*x* coordinate in pixels in 12 separate time bins ranging from 160 to 600 ms), horizontal movement (in pixels), and onset latency (ms) for the first microsaccade in a trial. Because the gaze was fluctuating during word reading, the differential gaze position relative to real words was used when analyzing DP effects in gaze position. This measure should better reveal the processing devoted to pseudo-words and non-words. Finally, to validate the relevance of the eye-movement findings in relation to actual reading skill, we calculated Pearson correlations between the gaze shifts (gaze position in each time bin and saccades) for text-reading speed.

## Results

### Response times

**Figure 5:**
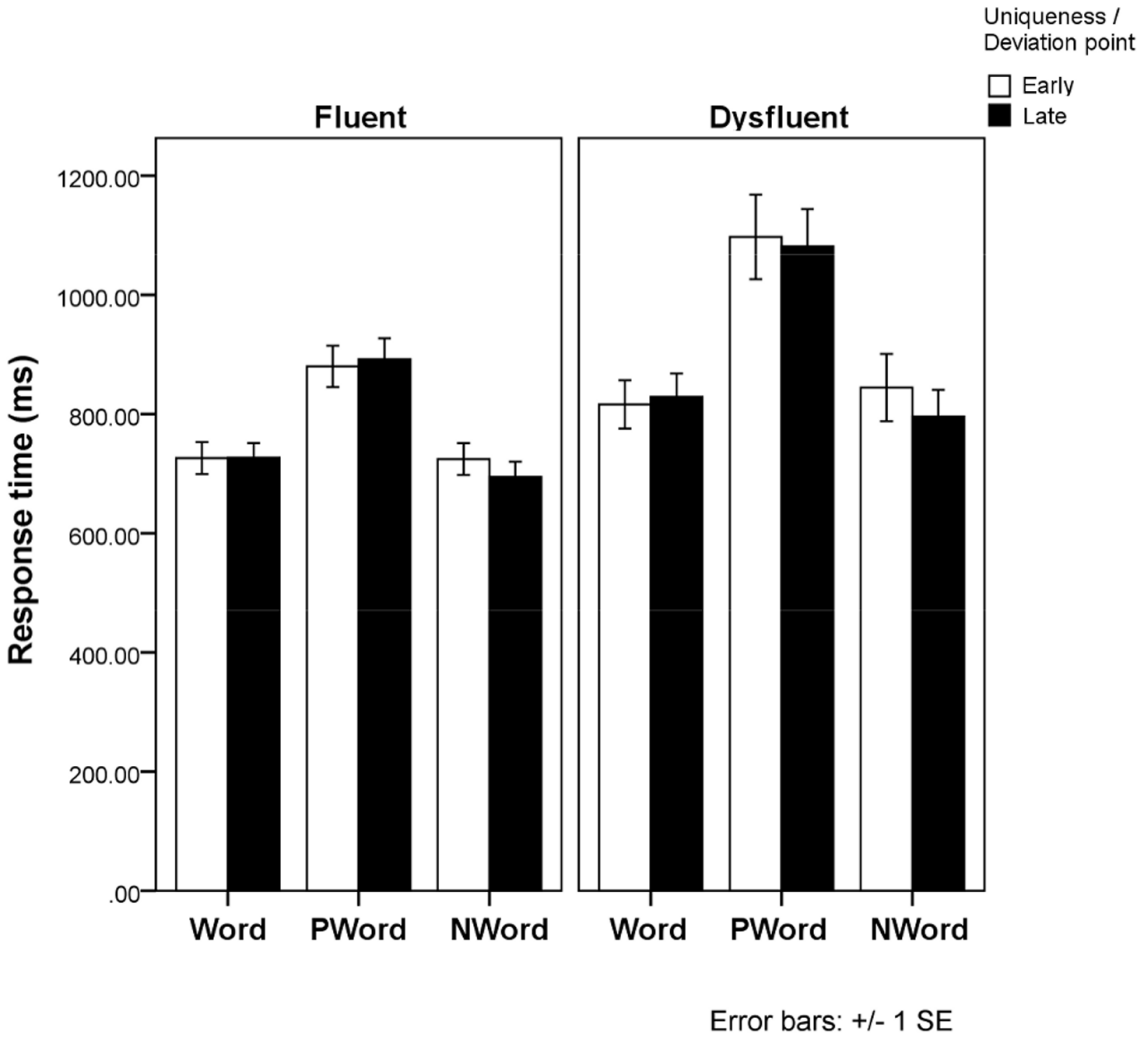
The influence of the word uniqueness point, and the pseudo-word and non-word deviation points, among fluent and dysfluent adult readers on lexical decision response times.

Figure 5 shows the mean response times. In word analysis, there was no effect of UP, or Group x UP interaction. Dysfluent readers generally responded more slowly than fluent readers, *F1*(1, 30) = 5.27, *p* = .029 and *F2*(1, 78) = 161, *p*<.001.

In pseudo-word and non-word analysis, the main effects of item types, *F1*(1, 30) = 253, *p*<.001, and *F2*(1, 170) = 341, *p*<.001, DP, *F1*(1, 30) = 6.48, *p* = .016, and groups, *F1*(1, 30) = 7.59, *p* = .010 and *F2*(1, 170) = 710, *p*<.001, were qualified by Group x Item type interaction, *F1*(1, 30) = 9.71, *p* = .004 and *F2*(1, 170) = 26.7, *p* = .001, which resulted from DYS readers responding relatively slower to pseudo-words. On average, items with late DP were responded to faster (867 ms) than items with early DP (888 ms).

### Gaze position

#### Words

**Figure 6:**
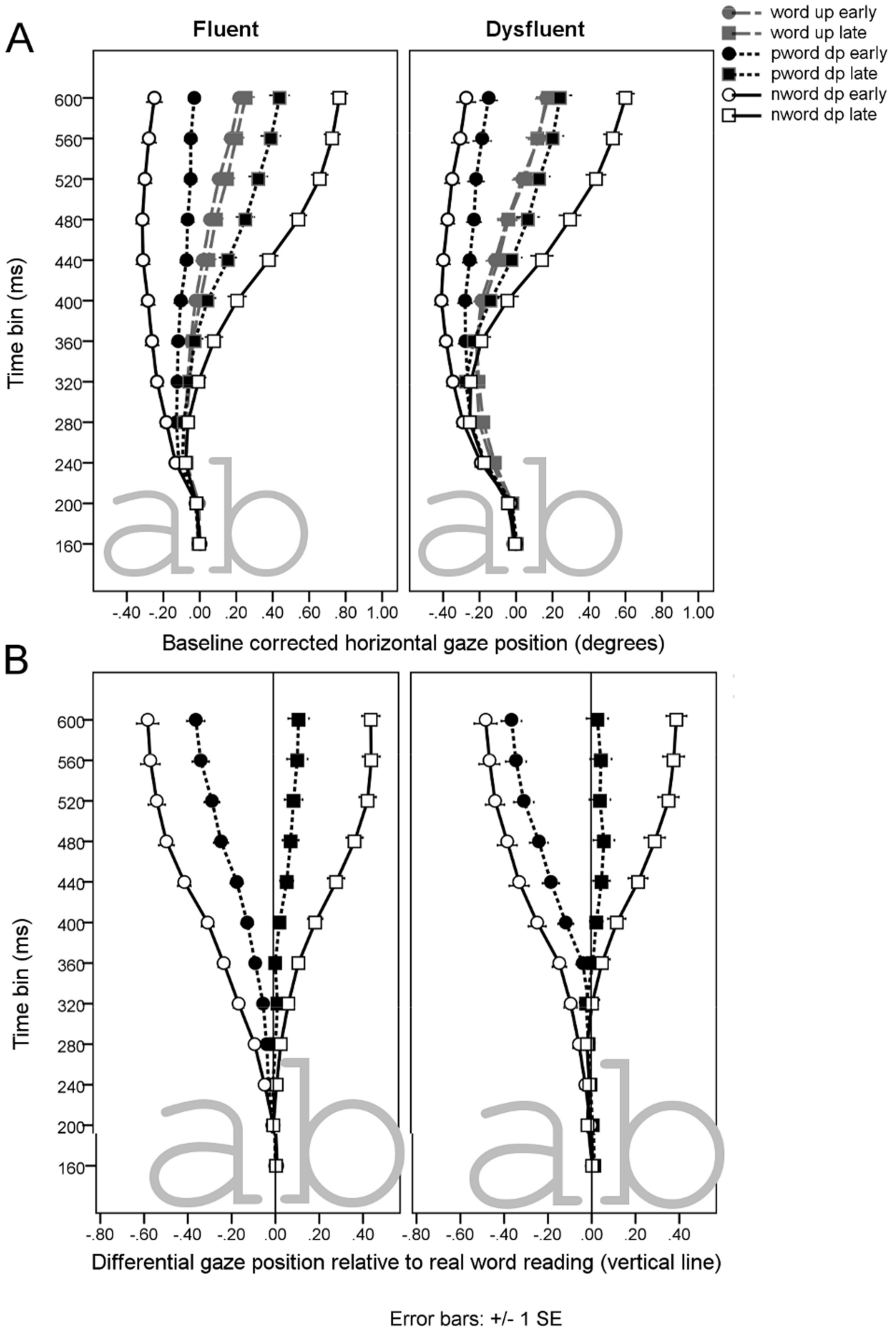
Gaze position data. Upper panels (A) illustrate baseline-corrected data and lower panels (B) illustrate differential real word minus pseudo- and nonword data.

Figure 6 A contains the mean gaze position for words in each time bin. There was no reliable effect of UP on real words. The gaze trajectory shows that the gaze position initially shifted toward the word beginning and later toward the word ending. The significant effect of groups in the time period of 280–440 ms was most significant in the time bin 320–360 ms, F(1,28) = 5.70, p = .024, indicating that the gaze of DYS readers was oriented more toward word beginnings compared to the FLUENT readers. At its largest, the gaze position for DYS relative to FLUENT readers was oriented 0.16 visual degrees (less than one-third the width of a letter) more toward the word beginning in the 360–400 ms time bin.

#### Pseudo-words and non-words

Table 3 summarizes significant results from the analysis of pseudo-word and non-word DP effects. Figure 6 B show the analyzed differential pseudo/nonword subtracted by word data, illustrating how the gaze position trajectories for early- and late-deviation points differ from fluctuations of gaze position during real-word reading. The main effect of DP from 240 ms onward shows that early- and late-DPs attracted gazes in the opposite directions. Early-deviating non-words started to affect gaze position at the 200–240 ms time bin, *t*(29) = −4.14, *p*<.001, and late-deviating non-words at the 240–280 ms time bin, *t*(29) = 2.41, *p* = .023. Early-deviating pseudo-words started to affect gaze position at the 240–280 ms time bin, *t*(29) = −2.57, *p* = .016, and late-deviating pseudo-words at the 400–440 ms time bin, *t*(29) = 3.32, *p* = .002.

**Table 3:** Pattern of significant ANOVA results of time bins for pseudo- and non-word gaze position data.

Effect	An.	200–240	240–280	280–320	320–360	360–400	400–440	440–480	480–520	520–560	560–600
Deviation point	*F1*	6.98*	24.7**	34.5**	48.4**	59.5**	95.5**	124**	175**	220*	227**
	*F2*	3.3^1^	7.3*	20.2**	59.7**	135**	223**	304**	349**	361**	362**
Item type	*F1*		4.73*	7.3*	3.2^1^				3.37^1^	11.2*	11.4*
	*F2*								3.2^1^	4.5*	3.9*
Dev* Group	*F1*		8.75*	10.3*	4.0^1^						
	*F2*		7.3*	18.1**	13.4**	6.8*	8.5*	17.2**	14.5**	17.6**	16.8**
Dev* Item type	*F1*		5.17*	30.0**	47.7**	87.2**	125**	126**	176**	131**	137**
	*F2*			10.3*	20.8**	34.0**	47.3**	55.5**	60.6**	50.0**	48.8**
Item type* Group	*F1*										
	*F2*								4.1*	5.88*	5.1*
Dev* Item type* Group	*F1*				3.02^1^	3.90^1^	4.9*	4.0^1^	3.52^1^		
	*F2*				2.87^1^	4.8*	8.2*	9.7*	7.6*	4.93*	

*p<.05,

**p< = .001,

1 *p*<.1.

Each cell contains an F-test value and its significance level. The degree of freedom values for all tests are 1, 28 for within-subject analysis (*F1*) and 1, 170 for item analysis (*F2*).

Two group-related interactions were significant: Deviation point x Group interaction relatively early in the time course (240–320 ms) indicated that the DP effect was larger in the FLUENT group than in the DYS group. The DP effect emerged earlier in FLUENT readers relative to DYS readers.: In the FLUENT group, early DP started to differ from words at the 200–240 ms time bin, *t*(17) = −4.32, p<.001, followed by the effect of late DP at the 280–320 ms time bin, *t*(17) = 2.58, p = .020. In DYS, the early DP started to affect gaze position at the 240–280 ms time bin, *t*(11) = −2.31, p = .017, whereas the effect of late DP appeared late, at the 400–440 ms time bin, *t*(11) = 2.82, p = .017.

Furthermore, late-appearing (400–440 ms) Item type x Deviation point x Group effects resulted from the FLUENT group showing increased responses in gaze position to early non-words DP than what was observed in the DYS group, F(1, 28) = 4.76, p<.038. This may result from DYS gazes being generally oriented toward word beginnings, which probably decreases the effect of early non-word DP in differential gaze position data.

#### Correlations

Pearson correlations between text-reading speed and gaze position were significant during the time period of 240–600 ms, indicating that the slower the reader, the more the gaze was oriented toward item beginnings. The correlation was strongest for late-deviating non-words at the 520–560 ms time bin, *r*(30) = 0.648, *p*<.001. The earliest correlation of gaze position to text-reading speed was also detected for late-deviating non-words at 200–240 ms, *r*(30) = .366, *p* = .039, whereas at the subsequent time bin (240–280 ms) the largest correlation between gaze position and reading speed was found among real words, *r*(30) = .518, *p* = .002.

### Microsaccadic and saccadic behavior

**Figure 7:**
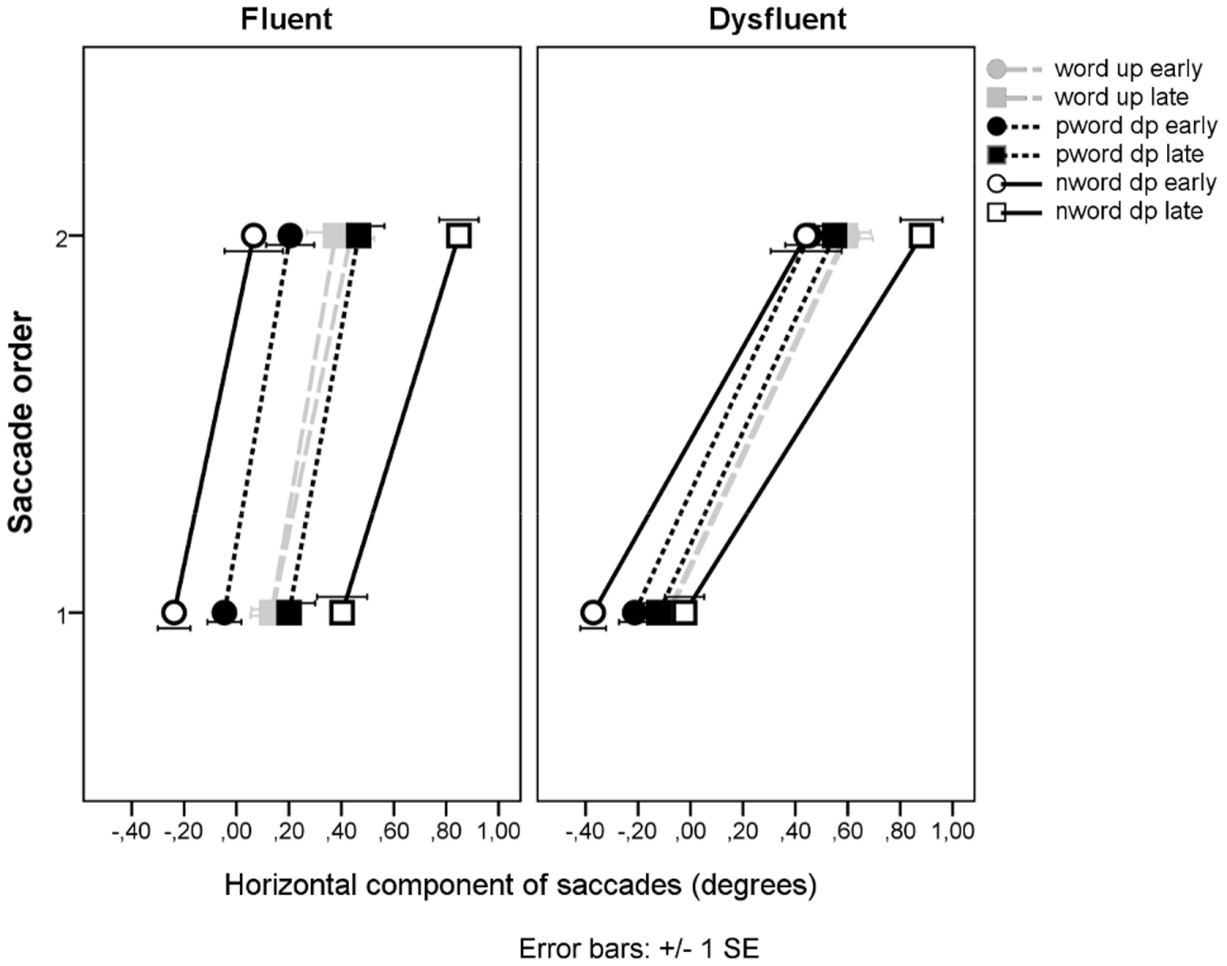
The horizontal movements of saccades.

Figure 7 shows the mean horizontal movement (relative to (micro-)saccade starting locations) of the first and second saccades in a trial. The word UP manipulation did not produce any significant effects in the saccade analysis.

Pseudo-word and non-word analysis of the horizontal component of the first saccade produced the following significant effects: Group, *F1*(1, 26) = 6.57, *p* = .017 and *F2*(1, 170) = 73.7, *p*<.001, deviation point, *F1*(1, 26) = 52.4, *p*<.001 and *F2*(1, 170) = 97.0 *p*<.001, interactions of Group x Deviation point, *F1*(1, 26) = 5.95, *p* = .022 and *F2*(1, 170) = 29.4, *p*<.001, and Item type x Deviation point, *F1*(1, 26) = 69.9, *p*<.000, and *F2*(1, 170) = 25.4, *p*<.001. In both groups, non-word DP had an overall larger impact on saccades than pseudo-word DP. However, in DYS readers, the late DPs affected the first saccade amplitudes less than in FLUENT readers.

Temporally, saccades for non-words were launched generally earlier, at the latency of 293 ms, than those for pseudo-words, which had a saccade at the latency of 329 ms, *F1*(1, 26) = 25.3, *p*<.001 and F2(1, 170) = 17.2, p<.001. Item type x Deviation point interaction, *F1*(1, 26) = 5.66, *p* = .025 and *F2*(1, 170) = 3.01, p = .084, reflected opposite effects in saccade latencies for pseudo-words and non-words. Saccades for early-deviating pseudo-words were launched 13 ms slower than those for late-deviating pseudo-words, whereas in non-words the pattern was reversed: early-deviating non-words received a saccade 14 ms earlier than late-deviating non-words.

**Figure 8:**
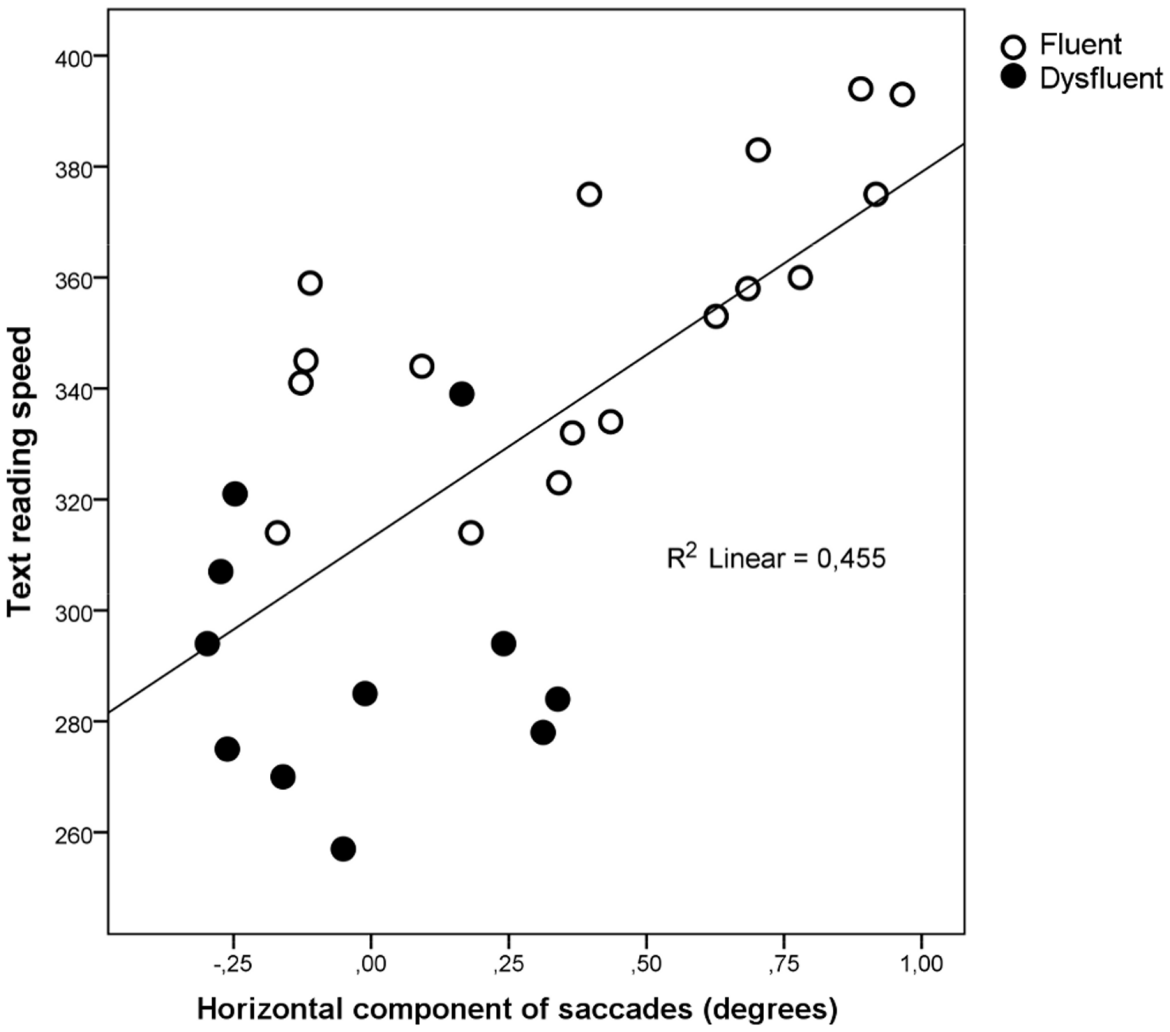
The correlation between text-reading speed (words in three minutes) and horizontal movement of the first saccade to late deviating nonwords.

Finally, the horizontal component of the first saccade was significantly correlated with text-reading speed. Figure 8 shows the scatterplot between text-reading speed (words in three minutes) and horizontal movement of first saccades to late-deviating non-words.

## Discussion

We examined whether response times, gaze positions and (micro-)saccadic eye movements would reveal serial within-word processing during visual word recognition among fluent and dysfluent readers. For this, the time course of gaze position shifts and saccadic latencies was compared between items with early versus late word uniqueness and pseudo/non-word deviation points. To study the processing levels in which this serial within-word processing occurs, we manipulated word UPs and pseudo- and non-word DPs in order to stress lexical, phonological, and orthographic levels of processing, respectively. We did not obtain any effects of word UP manipulation in response times or in eye-movement measures whatsoever, supporting the commonly accepted view that letters of familiar words are processed in parallel [14]–[17]; see [53] for recent empirical evidence.

Instead, the pseudo- and non-word DP manipulations strongly attracted gaze positions toward them, with early DP causing the gaze to shift to the left and late DP to the right. In line with the serial within-word processing hypothesis, items with early DP resulted in an earlier shift in gaze position than did items with late DP. The emergence of the orthographic non-word DP effects at 200–240 ms is well in line with brain activity being associated with orthographic processing starting at 150 ms after stimulus onset [18]–[22], when accounting for the time required for neural transmission from brain to eyes. Non-word DP effects on gaze position were closely followed by the effect of early deviating pseudo-words on gaze position (one time bin, 40 ms, later). This finding provides novel time-course evidence for the view that orthographic and phonological processing are closely tied in time, a view previously based mostly on indirect evidence gained through orthographic and phonological prime duration manipulations [17].

Despite our rather lenient yet representative criteria of reading problems in a transparent orthography, which is slow yet accurate reading, we were able to obtain some meaningful findings about reading fluency. Dysfluent readers' eye movements showed a pattern of results in line with our serial within-word processing hypothesis: that is, early DP would affect gaze position earlier than would late DP. Compared to fluent readers, the effect of early DP was delayed by only one time bin (40 ms) in dysfluent readers. Strikingly, the effect of late DP among DYS was delayed by three time bins (120 ms) in comparison to FLUENT readers. This large delay of the effect of late DP in eye movements suggests a deficiency in the early serial orthographic analysis of unfamiliar words. Theoretically, according to the visual attention span deficit hypothesis [54], dyslexic readers have a smaller visual span for letter recognition. The smaller span explains the dysfluent readers' slowness in detecting word-end cues, but it does not explain why such a deficit is present only when reading unfamiliar words. Therefore, the account most in accordance with the present gaze position data is that dyslexic readers have a deficiency in the early serial orthographic processing [16] of words that do not have solid orthographic memory representations. However, the impairment in early orthographic processing is clearly not only a deficiency in dysfluent readers' visual word recognition, but the highly delayed response times to pseudo-words suggest additional inefficiency in phonological-lexical search strategies [55]. Perhaps the dysfluent readers took additional time to determine whether our very word-like pseudo-words were instead real, but rare, words?

The fact that we did not obtain any word UP effect is in contrast with the inhibitory UP effects found in lexical decision and naming response times in English [44]–[46], but is in line with null results of an eye-movement study of reading [47]. There was an early tendency to shift gaze toward word beginnings, which is likely to result from prioritizing optimal vision for word beginnings: that is, the optimal viewing position, which is known to be a little left of word center [56]. However, the underlying reason for this prioritization may be that word beginnings are generally more important for lexical searches than are word ends [57]. Thus, it may be that when reading longer words extending over the human perceptual span, we would more likely find the effects of within-word predictability [4], [5], [13], such as the effects of word UP.

Response times to early-deviating pseudo- and non-word items were longer relative to late-deviating items, as previously discovered by Lindell and colleagues [45]. Thus, in contrast with our hypothesis, orthographic transparency seems not to invoke serial DP effects when measured in response times, despite the presence of serial DP effects in eye-movement measures. A potential explanation for this discrepancy is that response times may be more influenced by lexical processing whereas early eye-movement responses may reflect earlier and more mechanical decoding processes [58]. Also, speculatively, the effects in response times may resemble the word superiority effect: one can recognize letters faster when they are embedded in real words relative to random-letter strings [59]. Taking into account the early serial processing of unfamiliar words found in our data, word beginnings may activate representations more rapidly than do word endings, which, in turn, may provide faster top-down support for detecting anomalies at item endings.

The eye-movement results of pseudo-word DP effects were complex: the early-deviating pseudo-words had rather early and clear effects on gaze position, whereas the late-deviating pseudo-words had only a minimal effect. This seems to suggest that the mere point of divergence from other words in a language may not be responsible for the effect. Instead, it may be that sublexical units larger than bigrams drive eye movements. In line with this explanation, our early-deviating pseudo-words had somewhat lower initial trigram frequency (0.394 occurrences per 1000 words) compared to other initial, middle, and final trigram frequency values (1–1.5 occurrences per 1000 words) in pseudo-words. Despite being able to control initial bigram frequencies, we note that unique word beginnings are inherently tied to low larger-unit (trigrams and syllables) sublexical frequencies, and that it is beyond the scope of the present study whether such large-unit processing is orthographic, phonological or lexical by nature. Regardless, the present methodology seems to be well-suited for studying the time courses and processing levels of varying sizes of linguistic units.

In respect to theoretical accounts of visual word recognition, our results are in line with the dual-route account of reading, which suggests a parallel processing of letters when reading familiar short words [14]–[17] and serial within-word processing when reading novel words. In finding that the effect of non-word DP on gaze position shifts was serial by nature, we support the view that serial within-word processing stems from the orthographic processing stage, as postulated in the connectionist dual process model [14]. This leads to our results being incompatible with word recognition models that predict serial effects stemming from visual input or articulatory output processes [15] or solely from orthography-to-phonology mapping processes [14], [17]. We note that the question of serial vs. parallel processing is fundamental in cognitive science. For simplicity's sake, in the present paper serial effects are always assumed to result from serial within-word processing. We acknowledge that serial effects may result also from parallel letter processing if different letters are assumed to be processed at different speeds, as suggested by attention gradient models such as SWIFT [60].

In short, the present exploratory study demonstrates that even miniature eye movements sensitively reflect visual word recognition processes, more specifically serial within-word processing, as we were able to induce differences in the temporal-spatial trajectory of eye-movements by spatially manipulating subword-level information. In accordance with the general dual-route scheme of reading, our results revealed both parallel letter processing of known words and serial within-word processing of unfamiliar words [14], [16]. In addition, our results suggest that the processing of letters of unfamiliar words is serial at the orthographic processing level [16]. Dysfluent readers were especially slow in pseudo-word reading, tending slightly more toward word beginnings and showing a substantially delayed response to anomalies at pseudo- and non-word endings in gaze position. The delay in detecting word-end anomalies suggests an impairment in serial orthographic processing [16], whereas the phonological pseudo-word reading deficits are compatible with an additional deficit in lexical search processes [55].

### Methodological considerations

In this study, we introduced a novel way to analyze eye-tracking data by simply averaging raw gaze position signals. We also analyzed (micro-)saccades, which indeed were spatially oriented toward our DP manipulations. Temporally, saccades were launched by an average of 300 ms after item presentation; that is, soon after the earliest DP effects in gaze positions, but long before lexical decision response times. However, the saccade latencies of early- and late-deviating pseudo- and non-words showed conflicting and small effects (14 ms). It is clear that the determinants of saccade latencies during visual word recognition require further research. Gaze positions and (micro-)saccade analyses produced converging results, which is not surprising as the gaze position signals contained also saccadic eye movements. However, the gaze position signal was an even more sensitive measure of word recognition processes than were saccades, which suggests that the drift occurring during fixations may also be a signal carrying information of cognitive processing.

It must be noted that the results of our (micro-)saccade analysis are not directly comparable to microsaccade studies conducted in attentional cueing paradigms. Clearly, our data included eye movements of sizes of both microsaccades and saccades; we did not adopt the criterion of below-one-degree amplitude for microsaccades [10] because of the single distribution of saccadic amplitudes in our data (Figure 1), providing no opportunity to separate microsaccades from saccades. The somewhat larger within-word saccadic amplitudes in our study are probably due to our stimulus size: longer words require larger saccades. However, a large proportion of cases overlapped with the size of microsaccades (<1 degree), and these cases also showed the reported pattern of results.

Saccadic rates in our study (ranging from 3.1 to 5.4 hz across subjects) compare better with reading studies (∼4 hz) than with studies employing attentional cueing tasks (0.2–2 hz) [49]. In a comparable study to ours, Kuperman and Bertram [13] studied the recognition of complex compound words in lexical decision tasks. In their study, the average fixation duration was 262 ms, corresponding to a mean saccadic rate of>3 hz. Compared to attentional cueing studies, methodological reasons for high saccadic rates in our study include a lack of binocular eye movement criteria [1] and the fact that we did not analyze data during watching fixation cross prior to item presentation, during which eyes are mostly still, resulting in a low saccadic rate [1]. In conclusion, it seems probable that a large proportion of saccades in the present study are “voluntary” refixations [10], [11], [12]. Figure 4 shows a typical eye movement sequence during a single trial, illustrating the cognitive nature of our task: subjects more or less actively seek the point of anomaly in the stimuli. Taken together, the present study introduces new ways to analyze gaze position data during visual word recognition. Further work is needed for more detailed modelling of (micro-)saccades, but also for the possible cognitive signals carried by gaze drifts taking place between saccades and microsaccades.
